# Effects of a Rhythmic Drumming and Movement Intervention on Reaction Time in Community‐Dwelling Older Adults: A Nonrandomized Pilot Study

**DOI:** 10.1155/jare/3084489

**Published:** 2026-06-30

**Authors:** Austin S. Robinson, Alaine E. Reschke-Hernandez, Brittney Moshos, Peter Wright, Greg Walsh, Anne Graff, Carrie Ekins, Sarah Davey, Kiera Wilkinson, Nathan F. Johnson

**Affiliations:** ^1^ School of Music, University of Kentucky, Lexington, Kentucky, USA, uky.edu; ^2^ Department of Physical Therapy, University of Kentucky, Lexington, Kentucky, USA, uky.edu; ^3^ School of Sport, Nutrition and Allied Health Professions, Oxford Brookes University, Oxford, UK, brookes.ac.uk; ^4^ Drums Alive, Lexington, Kentucky, USA; ^5^ Drums Alive, Augsburg, Germany; ^6^ Center for Discoveries in Life Sciences, Coventry University, Coventry, UK, coventry.ac.uk; ^7^ Re:Cognition Health, Bristol, UK

## Abstract

Driving is often the preferred mode of mobility for older adults in the United States, yet age‐related functional declines increasingly compromise driver safety. Driver reaction time is one of the most critical factors related to accident avoidance and is particularly vulnerable to age‐related decline. Maintaining a physically active, cognitively engaging lifestyle can help attenuate age‐related declines in reaction time, and rhythm‐based exercise programs represent one such avenue. This pilot study examined preliminary evidence on whether a 10‐week Drums Alive program, a drumming‐based aerobic workout program, shows sufficient promise to maintain or improve reaction time for community‐dwelling older adults. Participants enrolled in the Drums Alive program were nonrandomly assigned to the intervention condition (mean age = 69.67 years, SD = 5.86). Twelve age‐ and sex‐matched participants from a previously published study served as the stretching comparison condition (mean age = 69.35 years, SD = 5.07). Simple reaction time (releasing accelerator), movement time (moving foot to brake), and total reaction time were recorded during a simulated brake‐onset task pre‐ and postintervention. There was a statistically significant intervention by time interaction for movement time, with Drums Alive participants showing a greater improvement (*M* = −0.054, SD = 0.061) compared to the stretching group (*M* = 0.006, SD = 0.044) and total reaction time, with Drums Alive participants showing a greater improvement (*M* = −0.080, SD = 0.089) compared to the stretching group (*M* = 0.059, SD = 0.165). No significant group differences were observed for simple reaction time. It is important to design music‐based interventions for a specific purpose to support client needs, enhance engagement, and align with evidence‐based practices. Our findings suggest that carefully designed music interventions that engage older adults in exercise may help maintain independence by improving driving‐related reaction time.

## 1. Introduction

Approximately one‐fifth of the population of the United States will exceed age 65 years by 2030 [1]. Generally, older adults in the United States prefer driving as the primary mode of transportation [2, 3]. Driving is a necessary part of daily life for many older adults, as it is a form of mobility that facilitates access to healthcare services, nutritional needs, and social activities [2, 3]. Public transit and other forms of individual transportation (i.e., bicycling and walking) are limited in many suburban and rural areas. City size, infrastructure, and safety measures contribute to the high number of vehicles on the road outside major cities [4]. Driving cessation is often associated with losing independence [5], a major concern of older adults [6]. Further, many older adults prefer to remain in their homes and engage in their communities [7, 8]. Thus, maintaining driving mobility is critical for preserving independence as Americans age.

Age‐related declines in mobility begin around age 40 years, with movements becoming slower and less coordinated [9, 10]. Adults 65 years and older experience declines in psychomotor speed, impacting their ability to evaluate their environment and execute an appropriate response [11, 12]. Such motor and cognitive skills are related to reaction time (RT), a proxy measure of cognitive–perceptual and sensory–motor skills [13]. RT indicates how quickly an individual can avoid a fall or stop a motor vehicle [14, 15]. Thus, preserving age‐related declines in RT is critical for maintaining the ability to react to environmental perturbations.

RT is the coalescence of an individual’s ability to perceive a stimulus, make a decision about the stimulus, and execute a motor response. At the most basic level, RT is comprised of two components, simple RT (sRT) and movement time (MT), both of which are affected by aging [14, 16]. sRT is indicative of cognitive acuity and central processing of external stimuli (e.g., noticing a light change from green to red) and is impacted by age‐related neurological changes in structure and function [17–19]. Processing environmental stimuli and planning a relevant response precede initiation and execution of a motor response, or MT (e.g., moving your foot from the accelerator to the brake pedal). Age‐related slowing in MT is related to neurological changes as well as structural and functional changes in skeletal muscle [20, 21]. Interestingly, physical fitness and activity may influence age‐related changes in RT by maintaining or improving cognitive and motor performance [22–25]. However, motivating individuals to move is a commonly reported challenge [26–28].

Music‐based interventions that induce total body rhythmic movements may motivate people to exercise and reinforce continuation of this health behavior [29]. Drums Alive (DA) is an aerobic exercise program that combines drumming, choreographed movements, and differential psychomotor responses to auditory and visual cues [30]. Golden Beats is designed for older adults, offering a low‐impact, highly‐adaptable version of the original program. This intervention is grounded in entrainment principles described by Trost and colleagues, which are evident in music novices as well as highly trained musicians [31]. For example, auditory patterns in music influence the firing rate of auditory and spinal motor neurons [32], which primes the motor system through anticipatory timing cues and patterns. The extent to which longer term changes are directly attributable to entrainment remains unknown. However, music can provide a temporal structure for motor planning and execution [31, 32] and motivate older adults to move, which may support the development of more efficient and coordinated motor patterns over repeated exposures.

Additionally, music often compels us to move (e.g., toe‐tapping, head bobbing, and dancing) and enhances our enjoyment of the experience [33, 34]. This “groove” phenomenon suggests that carefully selected music that prompts movement may support engagement, attentional focus, and repetition necessary for motor learning, even in individuals without formal musical training [32, 35, 36]. Thus, DA, which capitalizes on these principles, may optimize movement and promote older adult engagement in exercise [37].

Although rhythmic entrainment and music–movement coupling are well‐established neurobiological phenomena [31–36], and physical exercise is known to support cognitive and motor performance in older adults [22–25, 38], relatively few interventions have examined rhythm‐centered, tactile, whole‐body activities as a primary therapeutic approach for cognitive–motor outcomes in aging. Most existing music‐informed exercise approaches emphasize motivation, enjoyment, or general physical engagement rather than explicitly targeting sensorimotor timing and coordination processes through rhythmic movement [29, 37]. Moreover, unlike digital and computerized cognitive training, DA engages embodied, multisensory motor processes that may support improvements in functional movement. Empirical investigations of active drumming interventions and their effects on functional RT outcomes in older adults, therefore, remain limited.

The aim of this nonrandomized pilot study was to gather preliminary evidence on whether DA shows sufficient promise as a therapeutic intervention for older adult RT to warrant future investigation. We did not include a priori hypotheses or power calculations since this was a pilot study [39]. To that end, we compared RT outcomes (sRT, MT, and tRT) between participants who completed the DA intervention and a passive stretching intervention without music via a simulated brake‐onset test, with findings intended to inform the design of future hypothesis‐driven trials.

## 2. Materials and Methods

### 2.1. Participants

The Institutional Review Board at the University of Kentucky approved this study. Participants were recruited from November 2018 to February 2019 via a local newspaper advertisement, fliers posted on the University campus, and at the Senior Center in Lexington, Kentucky, where study activities also occurred. Baseline data collection was completed in February 2019, the intervention was conducted from March through May 2019, and postintervention assessments were completed in May 2019.

For this pilot investigation, participants were included if they regularly visited the Lexington Senior Center and were at least 60 years old. We excluded candidates who (a) had an expired driver’s license; (b) had not driven a motor vehicle in the United States in the last 6 months; (c) had a medical condition that prevented them from completing the tasks; (d) were under the influence of alcohol or other recreational drugs; (e) were taking any medication that may cause drowsiness or prohibit them from operating large machinery; (f) had any anthropometric measures that might threaten subject or research personnel safety while on testing equipment or during test administration (e.g., equipment use that may have height or weight restrictions); (g) were not Fayette County residents; (h) did not sign the liability waiver; (i) did not preregister for the Drums Alive class at the local Senior Center; (j) were not independent or accompanied by a caregiver; (k) were not age 60 years or older, or the spouse of a county resident age 60 or older; or (l) were not oriented in time or place.

In addition to the criteria described above, participants were required to demonstrate sufficient cognitive and neurological function to safely complete all study tasks. Cognitive screening included the Mini‐Mental State Exam (MMSE), digits forward and backward, Trail Making Tests A and B, Operation Span (O‐Span), Symmetry Span Test, Brief Repetitive Thought Scale, and the Dresden Spatial Navigation Task. Participants with cognitive impairments that prevented safe participation were excluded. Although no formal neurological exam was conducted, participants were required to be free from known neurodegenerative conditions, recent stroke, or significant tremors that could impair motor performance, based on self‐report and review of medical history. Vision screening was conducted by confirming participants had sufficient visual acuity to safely interact with the driving simulator, with corrective lenses allowed if normally used. These additional criteria ensured that participants were able to safely perform both the Drums Alive intervention and driving simulation tasks.

Participants were initially recruited through flyers distributed at the community senior center and via local advertisements. Interested individuals contacted the research team and completed a prescreen phone interview to determine eligibility based on inclusion/exclusion criteria, including age, driving history, cognitive and neurological status, and medical safety. Eligible participants were scheduled for a preintervention assessment session at the university, lasting approximately 90 min, which included cognitive testing, motor assessments (HuMAP, Timed Up and Go, Chair Stand, and Sitting Rising Test), and one round of the driving simulator.

During pretesting, all participants received verbal and written explanations of the study’s purpose, procedures, potential risks, and benefits. They were informed of their right to decline or withdraw from the study at any time without consequence. Each participant confirmed their understanding and voluntarily agreed to participate by signing a written informed consent document approved by the Institutional Review Board.

Following pretesting, Drums Alive participants completed the 10‐week intervention, delivered twice per week for one hour per session, at the senior center. Within 2 weeks of completion of the intervention, participants returned to the university for a postintervention assessment using the same battery of tests. All assessments and intervention sessions were conducted by trained research personnel under standardized procedures to ensure consistency and safety.

Participants were nonrandomly assigned to study conditions based on self‐selection; those who had preregistered for the Drums Alive class at the Lexington Senior Center were enrolled in the DA intervention condition, while age‐ and sex‐matched participants from a previously published study [40] comprised the stretching comparison condition. It should be noted that the two cohorts were recruited through distinct processes and screened on partially nonoverlapping eligibility criteria. Notably, DA participants were required to hold an active driver’s license and report recent driving history, criteria not required of the Johnson et al. (2021) stretching cohort. Conversely, Johnson et al. (2021) participants were required to meet MRI compatibility standards and obtain physician clearance prior to participation, and completed a maximal graded exercise test before beginning the stretching protocol. These procedural and eligibility differences between cohorts represent a potential source of confounding and should be considered when interpreting between‐group comparisons. Full details regarding recruitment and eligibility procedures for the stretching comparison group are reported in that publication.

**FIGURE 1:**
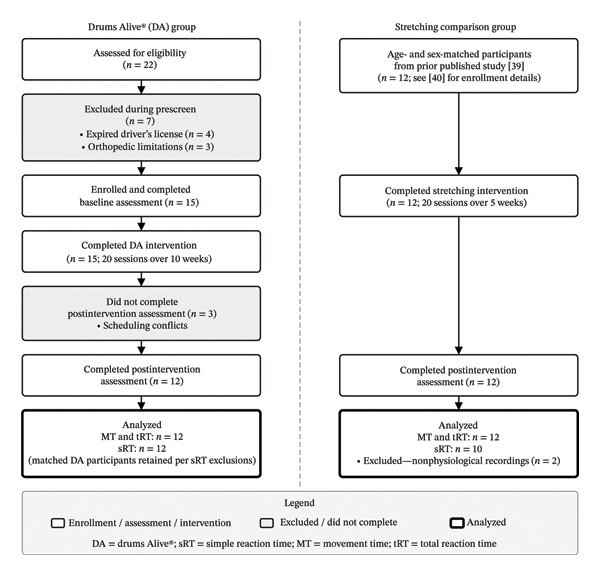
Participant flow diagram.

An initial pool of 22 individuals completed the prescreening process, with four being ineligible due to expired driver’s licenses and three being ineligible due to orthopedic limitations. A total of 15 community‐dwelling older adults enrolled in the Drums Alive intervention (Caucasian = 15, male = 2) and were instructed to complete the baseline assessment, full Drums Alive intervention, and postintervention assessment (mean age = 69.67 years, SD = 5.86, range = 62–79). Three participants did not complete the driving simulator task following the intervention due to scheduling conflicts following the intervention. Twelve age‐ and sex‐matched participants [40] were included for the stretching comparison and all measures (mean age = 69.35 years, SD = 5.07, range = 62–77) (refer to the participant flow diagram in Figure 1).

Due to the nature of the interventions, masking of participants and intervention deliverers was not possible. Outcome assessors were not masked to group assignment during data collection and analysis. No formal strategies to increase compliance or adherence were employed in either condition. Tracking of recruitment, eligibility, and retention provides preliminary feasibility data for future studies.

### 2.2. Independent Variable

#### 2.2.1. DA Intervention

Authors 1 and 6 led the DA intervention. Author 1 is a board‐certified music therapist with a master’s degree and 8 years of clinical experience. Author 6 is a licensed occupational therapist with a master’s degree and over 30 years of clinical experience. The group intervention consisted of two one‐hour sessions per week (20 sessions total) across 10 weeks, delivered live and in person at the Lexington Senior Center. All sessions followed a protocol and consisted of drumming and movement sequences to target cognitive–perceptual and sensorimotor function [30]. Participants were provided with a large‐diameter stability ball that served as the drum, a bucket that acted as a riser and holder for the stability ball, and two drumsticks.

Participant‐preferred music selections for the sessions were identified through a brief interview conducted during the preintervention assessment. Participants were asked to list songs or genres they regularly listened to and enjoyed. These selections were incorporated into approximately 30% of the intervention sessions, with the remaining music consisting of instructor‐selected novel tracks (50%) and familiar popular selections (20%). This approach ensured that participants were engaged while maintaining consistency across sessions. Music preferences were diverse but clustered around rock (e.g., Elvis Presley, Queen), Pop (e.g., Pharrell Williams, Justin Timberlake), and dance (e.g., ABBA, The Rumbar).

Each DA session had three phases. Each phase combined drumming, active music listening, and movement. Phase 1 started with a 15‐min warm‐up focused on stretching and breathing (approximately 4 min) and fine and gross movements of upper and lower extremities (approximately 10 min). Participants used one drumstick in each hand to practice bilateral fine motor skills. Instructors used high‐groove music selections (e.g., “Uptown Funk” by Bruno Mars) to structure warm‐up movements and increase engagement. Phase 2 comprised 35 min of higher intensity choreographed movements and rhythmic drumming patterns. Musical selections typically featured a faster tempo with a highly salient beat to support in‐the‐moment rhythmic entrainment and movement synchronization (e.g., “El Timbal” by The Rumbar). Phase 3 involved a 10‐min cool‐down focused on mindfulness, breathing, and stretching with slower musical selections (e.g., “Nara” by Tim Godfrey) to structure and enhance exercise recovery.

In addition to the structured drumming and movement sequences, participants were exposed to a visual cue component in which images were displayed on a screen corresponding to thematic music selections. This was based on the DA protocol, which uses visual aids for cues. For example, images of different fish or birds represented different drumming or movement patterns that were executed during the songs “Under the Sea” and “Rockin’ Robin,” respectively. Participants were instructed to alter their drumming or movement patterns in response to the images, providing an integrated cognitive–motor challenge that required attention, pattern recognition, and coordination. This reinforced the connection between visual cues, musical rhythm, and motor response.

#### 2.2.2. Stretching Comparison

**FIGURE 2:**
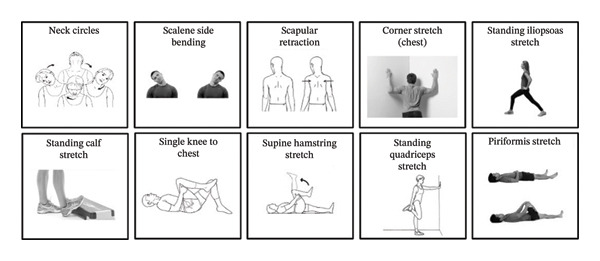
Representative stretching exercises. Participants were instructed to hold all stretches for a minimum of 30 s (maximum of 60 s) for three sets. Isometric holds were held for 5 seconds. Ten additional stretches were offered as progressive or alternative options (not pictured).

The details for the stretching intervention, including setting, demographics, and procedures, are not reported in the current manuscript; readers are referred to the previously published work for available procedural details [40]. A licensed physical therapist designed the stretching comparison to target common postural impairments in older adults. Like the DA intervention, the stretching comparison consisted of 20 one‐hour sessions; however, it occurred three times per week across five weeks. Doctor of physical therapy professional students delivered all stretching sessions. Each stretching session had two phases: (1) a 10‐min warm‐up on a recumbent bike and (2) 50 min of total body stretches and targeted isometric holds. Instructors guided participants to hold all stretches for a minimum of 30 s (maximum of 60 s) and complete three sets. Isometric holds were 5 seconds. Figure 2 illustrates the stretches. Progressive or adapted options were offered for 10 stretches (not pictured).

The stretching comparison condition was selected because it provides a structured, time‐matched physical activity condition without rhythmic engagement, active music listening, or the multisensory demands characteristic of the DA intervention, thereby allowing for isolation of the active ingredients of the experimental condition [38, 41]. Although Phase 1 of the DA intervention included brief stretching as part of a full‐body warm‐up sequence, this component was approximately 4 minutes in duration, determined by the length of a single song, and was functionally distinct from the structured, total‐session passive stretching protocol used in the comparison condition. Importantly, no adverse events or unintended effects were observed in either group during the intervention period.

#### 2.2.3. Driving Simulator

**FIGURE 3:**
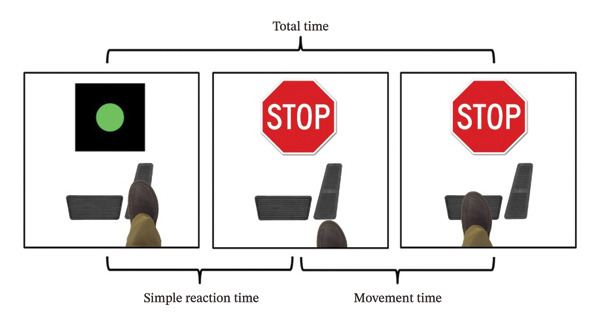
Brake‐onset reaction time test. Participants were instructed to remove their foot from the accelerator (simple reaction time; sRT) and press the brake (movement time; MT) as soon as they perceived the stop sign. The sum of sRT and MT represents the total reaction time (tRT) of the brake onset test.

A computerized driving simulation task was used to measure sRT, MT, and tRT on a brake‐onset task at baseline and postintervention (STISIM Drive, Systems Technology Inc., Hawthorne, CA). The driving simulator was located in an 8′ × 15′ windowless room in a back hallway to limit distractions. Participants were instructed to fully depress the accelerator to begin the trial, which triggered the presentation of a green light on the computer monitor. Participants were asked to fully depress the accelerator until the green light changed to a stop sign. At this point, participants were instructed to remove their foot from the accelerator (sRT) to depress the brake (MT). Participants practiced this maneuver three times, were asked if they had any questions, and then completed five consecutive trials [42]. The mean of all recorded trials was used in all analyses. Green light duration varied across all trials at chance occurrence, such that green‐to‐red transitions fell within a minimum of 3 seconds and a maximum of 8 seconds. Figure 3 illustrates the requisite action for sRT and MT (sRT + MT = tRT).

### 2.3. Statistical Analysis

A two‐way (time × condition) between‐subjects ANOVA was conducted using SPSS 28 software (SPSS Inc., Chicago, IL, USA) to assess the impact of DA relative to the stretching comparison for change in sRT, MT, and tRT (all in seconds) from baseline to postintervention. Given the pilot nature of this study, a per‐protocol analytic approach was used, in which only participants who completed both the intervention and postintervention assessment were included in analyses. Demographic equivalence between groups was addressed by design through age‐ and sex‐matching rather than formal statistical testing, consistent with the pilot nature of this study.

To ensure data accuracy, we adopted a data‐cleaning procedure that removed data that was nonphysiologic in nature, defined as a recorded value of zero for sRT, MT, or tRT. Two participants in the stretching group fit this description and were removed from all subsequent analyses (*n* = 10). Participants were removed as zero values were recorded across multiple trials within the postintervention session. Thus, these testing sessions were defined as artefacts and removed from subsequent analyses. The two age‐ and sex‐matched DA participants were included in all analyses (*n* = 12). Importantly, the removal of the two DA participants did not change the outcomes of the analysis. Given the small sample size and exploratory nature of this study, no covariates beyond age and sex matching were included in the analysis. Formal covariate adjustment was not employed because the assumptions required for ANCOVA are more difficult to satisfy in small samples, which reduces statistical power and validity. Age and sex equivalence between groups was addressed by design through matching rather than statistical control. No correction for multiple comparisons was applied, consistent with the exploratory intent of this study and the theoretical relatedness of the three outcome measures, which are all components of the same simulator‐measured RT construct. Regarding statistical assumptions, ANOVA is robust to moderate violations of normality, particularly in the context of small pilot samples. Shapiro–Wilk testing indicated no violation of normality for MT in the DA group (*p* = 0.489), with only mild skew observed in the stretching group (*p* = 0.045). No indication of assumption violations was observed across outcomes.

## 3. Results

The following results are presented as preliminary and exploratory observations consistent with the pilot nature of this study. Between‐group comparisons are intended to inform future trial design and assess the feasibility of the study’s methodology rather than draw definitive conclusions about the effectiveness of DA relative to stretching. Effect sizes are reported for all outcomes to provide preliminary estimates for sample size planning in future powered investigations.

sRT did not differ significantly between the DA and stretching conditions at baseline, MD = 0.028 s, *p* = 0.236, 95% CI = −0.020, 0.075. We did not find a main effect of time (*F* (1, 20) = 0.396, *p* = 0.550, *η*_*p*2_ = 0.018) or condition (*F* (1, 20) = 0.145, *p* = 0.708, *η*_*p*2_ = 0.007) on sRT, and there was no significant interaction (*F* (1, 20) = 3.107, *p* = 0.093, *η*_*p*2_ = 0.134; sRT change for DA group, *M* = −0.026 s, SD = 0.065, 95% CI = −0.089, 0.037; and for stretching, *M* = 0.053 s, SD = 0.140, 95% CI = −0.016, 0.123).

**FIGURE 4:**
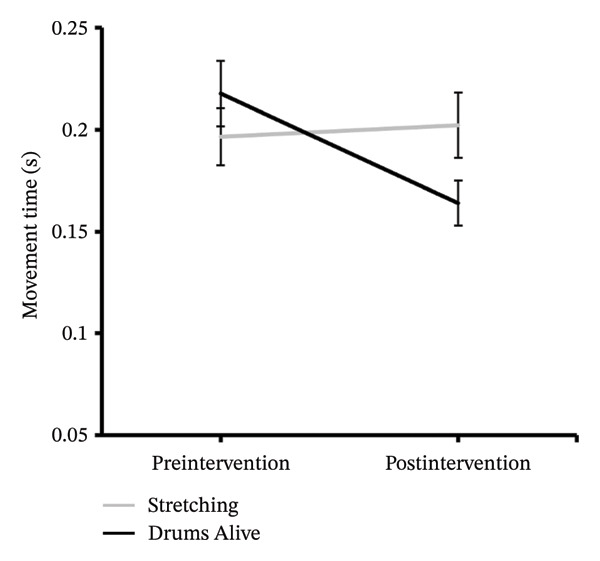
Brake‐onset movement time as a function of condition and time. The DA group demonstrated a significant decrease in MT from baseline to postintervention (*M* = −0.054, SD = 0.061). In contrast, the stretching group did not significantly change from baseline to postintervention (*M* = 0.006, SD = 0.044). Error bars represent the standard error of the mean.

MT did not differ significantly between the DA and stretching conditions at baseline, MD = 0.022 s, *p* = 0.331, 95% CI = −0.024, 0.067. There was no main effect of time (*F* (1, 20) = 4.27, *p* = 0.052, *η*_*p*2_ = 0.176) or condition (*F* (1, 20) = 0.248, *p* = 0.624, *η*_*p*2_ = 0.012) on MT. However, as illustrated in Figure 4, we found a significant interaction between condition and time (*F* (1, 20) = 6.679, *p* = 0.018, *η*_*p*2_ = 0.250). Pairwise comparisons revealed that participants in the DA group demonstrated a significant decrease in MT from baseline to postintervention (*M* = −0.054 s, SD = 0.061, *p* = 0.003, 95% CI = −0.086, −0.021). By contrast, those in the stretching group did not demonstrate a significant change in MT (*M* = 0.006 s, SD = 0.044, *p* = 0.730, 95% CI = −0.030, 0.042).

**FIGURE 5:**
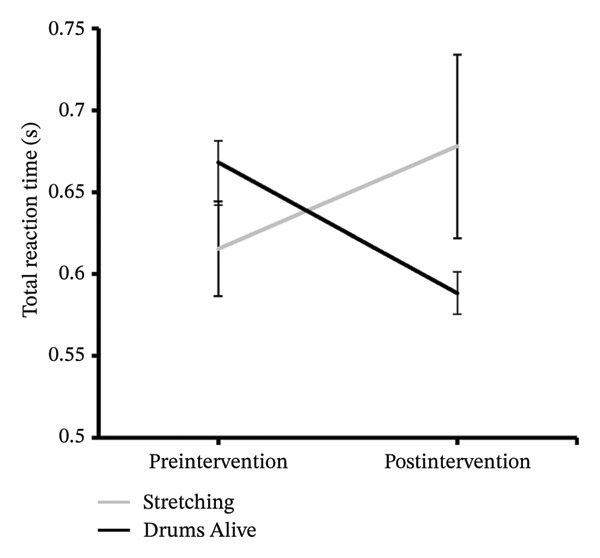
Brake‐onset total time as a function of condition and time. The DA group demonstrated a significant decrease in tRT from baseline to postintervention (*M* = −0.080, SD = 0.089). In contrast, the stretching group did not significantly change from baseline to postintervention (*M* = 0.059, SD = 0.165). Error bars represent the standard error of the mean.

tRT did not differ significantly between the DA and stretching conditions at baseline, MD = 0.049 s, *p* = 0.221, 95% CI = −0.032, 0.131. There was no main effect of time (*F* (1, 20) = 0.139, *p* = 0.713, *η*_*p*2_ = 0.007) or condition (*F* (1, 20) = 0.219, *p* = 0.645, *η*_*p*2_ = 0.011) on tRT. However, as illustrated in Figure 5, there was a significant interaction between condition and time (*F* (1, 20) = 6.329, *p* = 0.021, *η*_*p*2_ = 0.240). Pairwise comparisons revealed that participants in the DA group demonstrated a significant decrease in tRT from baseline to postintervention (*M* = −0.080 s, SD = 0.089, 95% CI = −0.157, −0.002), and those in the stretching group did not demonstrate a significant change in tRT (*M* = 0.059, SD = 0.165, 95% CI = −0.026, 0.144).

## 4. Discussion

Although pilot study results should be interpreted cautiously [39], we aimed to collect preliminary evidence on whether a rhythmic drumming intervention (DA) shows sufficient promise to improve RT in older adults to warrant future investigation. RT (sRT, MT, and tRT) was captured via a simulated brake‐onset task before and after a 10‐week DA intervention and compared to a 5‐week stretching intervention. Our preliminary observations suggest that this novel music‐based intervention may be feasible for older adults in a community setting. We observed a significant improvement in MT and tRT for the DA group but did not observe a significant change in the stretching group. No change was observed in sRT across either group.

### 4.1. Interpretation of Findings

#### 4.1.1. Movement Time and Musical Rhythm

Age‐related declines in psychomotor function include slower movements, resulting in reduced accuracy, control, and movement coordination [9, 10]. Findings of this pilot study demonstrate that older adults may improve functionally relevant MT through drumming‐based aerobic exercise and can inform subsequent randomized controlled trials. Further, stretching may serve as a feasible physical activity comparison condition, as it provided a structured, total time‐matched intervention without rhythmic or music‐based elements. These observations align with the suggested role of pilot studies to test trial design and intervention delivery, as well as to design and test transfer of skills from an intervention to functional tasks [39, 43, 44], vis‐à‐vis movement gains to functional tasks. Key features of the DA intervention may help explain findings of this study and inform future research.

Moving to music is a common human experience involving widespread bilateral brain activation [32, 45]. This response is driven by our auditory system’s ability to detect rhythmic patterns in sound [34]. DA capitalizes on the therapeutic application of time‐based musical elements (i.e., rhythm, beat, and tempo) to optimize movement [33, 34]. In older adults without formal musical training, these rhythmic cues may have facilitated sensorimotor synchronization and temporal prediction, which may have supported more efficient and coordinated movements during exercise [34, 36, 45]. Further, the use of faster music with a clear, driving beat may have required higher intensity movements and supported rhythmic coordination during training. Over repeated sessions, rhythmically structured, cognitive–motor practice may engage motor‐related neural networks, including the basal ganglia, supplementary motor area, and premotor cortex [34], which are critical for motor planning and control [45]. Although this pilot study suggests DA may be an effective intervention for the older adult population, future mechanistic studies are warranted to confirm potential training‐related effects in these motor control regions.

Drumming was embedded throughout each DA session in the study. Drumming is a high‐energy activity [46] that can be readily tailored to individuals with varying musical expertise [47]. It provides a salient visual target (e.g., the drum) and structured temporal cues (e.g., rhythmic music), which can support coordinated movement patterns and pacing. Drumming also promotes aerobic conditioning through total body movements at varying intensity levels [30, 46]. Further, participants receive multimodal sensory input, including auditory and vibrotactile feedback through drumstick impact [32, 34]. During music‐supported activity, these rhythmic and sensory cues may facilitate sensorimotor synchronization and increase movement repetitions. Over repeated sessions, engagement in rhythmically structured, whole‐body movement may contribute to improvements in MT, coordination, and attentional readiness. However, the specific mechanisms underlying these potential transfer effects remain to be elucidated; large‐scale, rigorous clinical trials are needed to evaluate the health effects of participating in a DA program.

#### 4.1.2. Transfer of Music Engagement to Reaction Time

Specificity regarding the type and design of a music intervention is critical to optimizing transfer from training to a functional task [48]. Intervention design may explain why we did not observe an effect of DA on sRT. As previously discussed, the DA intervention largely consisted of drumming and movement to recorded music with qualities that potentially influenced the urge to move and supported movement dynamics (i.e., timing, velocity, and force) [32, 34, 36]. The group leaders also incorporated tasks that relied on short‐term memory, alternating attention, and pattern recognition (e.g., play a specific drumming pattern when you see a picture of a fish or execute a specific movement pattern when you see a picture of a shark). Nonetheless, exercises specifically targeting psychomotor speed typically comprised only 5 min of each hour‐long session. Further, the rhythmic components of the intervention offered more predictability and structure than encountered in a complex and rapidly changing real‐world situation such as driving.

Another plausible explanation for our results may be the discrepancy between the outcome measure and the intervention. Most DA sessions focused on auditory pattern recognition and response (e.g., rhythmic pattern call‐and‐response) or visual tasks that included auditory and anticipatory cues (i.e., inherent repetition and predictability in music). These activities are quite different from the simulated brake test’s pure visual processing task. Auditory processing is faster and more accurate than visual processing [32, 36], and the driving simulator used to evaluate RT relied entirely on visual cues. As such, our nonsignificant observation may reflect a combination of the DA intervention’s design and the simulator’s utility to evaluate the benefits of this intervention. Future studies would benefit from incorporating an auditory cued RT task alongside visually cued measures to more directly evaluate the transfer of auditory–motor training to functionally relevant outcomes.

The effect sizes observed in this pilot study provide preliminary estimates to inform the design of future powered investigations. The interaction effects for MT (*η* ^2^ = 0.250) and tRT (*η* ^2^ = 0.240) reflect large effects, while the interaction effect for sRT (*η* ^2^ = 0.134) reflects a medium effect using conventional benchmarks. Although these estimates should be interpreted cautiously, they suggest that DA may produce meaningful changes in movement‐related RT outcomes and provide a basis for sample size planning in future hypothesis‐driven trials. Future studies with larger, more diverse samples and randomized designs are warranted to determine whether these preliminary effect sizes are replicable and to more precisely estimate the magnitude of DA’s impact on older adult RT.

Collectively, these findings should be interpreted in the context of several methodological considerations. Although the total number of sessions was equivalent across conditions, the DA and stretching interventions differed in their temporal distribution, with DA delivered over 10 weeks at two sessions per week and stretching delivered over 5 weeks at three sessions per week. Distributed practice schedules with longer intersession intervals have been associated with superior motor memory consolidation and long‐term retention compared with more condensed practice schedules [49], suggesting that the spacing of the DA intervention may have contributed to the observed improvements in MT and tRT, independent of rhythmic content or total dosage. Future research should control for session frequency and intervention duration to isolate the effects of rhythmic exercise from potential advantages of distributed practice. Furthermore, the following limitations should be considered when interpreting these findings.

## 5. Limitations and Conclusion

The small sample size of this pilot study limits generalizability, and our observations should be considered preliminary evidence and interpreted with caution. Additionally, the chosen sample size of 15 community‐dwelling older adults prioritized feasibility over power, limiting the generalization of our results. While the number of sessions was consistent between groups, the DA and stretching interventions differed in duration (10 vs. 5 weeks) and frequency (2 vs. 3 times per week). Although the stretching condition served as a feasible, total time‐matched comparison, future hypothesis‐driven trials should design the comparison to isolate specific intervention mechanisms (e.g., multisensory cues, rhythmic entrainment, etc.). Participants were nonrandomly assigned to the condition based on self‐selection, which introduces a potential source of bias and further constrains interpretation of the results. Participants were nonrandomly assigned to the condition based on self‐selection, which introduces a potential source of bias and further constrains interpretation of the results. Furthermore, the stretching comparison condition was drawn from a historically separate cohort with distinct recruitment procedures and eligibility criteria. Differences in preintervention screening demands, including MRI compatibility requirements, physician clearance, and a maximal graded exercise test completed by the participants in Johnson et al. (2021) prior to stretching, may have introduced systematic differences between cohorts that are not fully accounted for in the current analyses. These cohort differences further constrain the interpretation of the between‐group comparisons. Group assignment was not anonymous to outcome assessors in the current study, which may introduce bias. However, because the primary outcomes were recorded objectively via a computerized driving simulation, the risk of assessor scoring bias in the reported RT outcomes is minimal. Future trials should ensure that group assignment is not disclosed to outcome assessors, with analysis conducted without knowledge of condition assignment to minimize potential bias. Nonetheless, we gathered meaningful information regarding the potential impact of the DA intervention on older adults.

The small convenience sample of predominantly white female older adults from a midsize city limits the generalizability of findings to the broader population of older adults. Individuals with diverse cultural and ethnic backgrounds may respond differently to the DA intervention, highlighting the critical need for greater diversity in research samples. Importantly, this lack of diversity represents a significant gap in music [50] and aging research [51, 52]. Furthermore, emerging research indicates that sex and gender may influence cognitive aging [9, 52], underscoring the importance of recruiting a diverse, balanced sample to understand the intervention’s potential effects. Additionally, data were collected at a single community senior center in a midsize city, which further limits the generalizability of the findings across geographic, cultural, and institutional contexts. Future investigations should prioritize recruitment strategies that deliberately engage underrepresented populations across multiple community sites to improve the diversity and generalizability of findings.

Finally, we did not consider several participant characteristics that may impact our results. Although participants could safely engage in DA and stretching, we did not document their baseline or postintervention fitness levels. Future studies should measure cardiorespiratory fitness as it is related to sRT [42]. Future studies should also assess participants’ musical expertise or rhythmic skill. Although basic sensorimotor entrainment is expected across older adults, individual differences in rhythmic ability may influence the degree of benefit from the DA intervention. Rhythmic expertise has been linked to cognitive processes such as inhibitory control, attention, reading, and language skills [53]. Future research could evaluate whether beat‐keeping or rhythmic skill moderates responses to the DA intervention. Importantly, although musical experience influences the auditory system, such that trained musicians perform better on tasks such as speech‐in‐noise [54], our intervention was not designed to replicate this level of auditory training. Given that older adults commonly experience hearing loss [55], future studies could still include baseline music and hearing assessments to characterize participants and explore whether rhythmic music‐based interventions indirectly support auditory attention or perceptual engagement.

In conclusion, DA’s physical and cognitive demands may provide an engaging form of exercise for older adults. Preliminary findings suggest that DA’s combined aerobic and musical components may have meaningfully influenced driver‐related MT and tRT relative to a passive stretching comparison condition. A key question in supporting older adults’ health, mobility, and independence is how to motivate older adults to exercise regularly. Our findings suggest that carefully designed music interventions may promote older adult engagement in exercise. Future collaborative research on DA with a larger and more diverse sample of older adults is warranted to help us understand DA’s cognitive, physical, and social benefits in holistically supporting our community members as they age.

## Funding

This study was supported by the National Institutes of Health CTSA UL1TR000117.

## Disclosure

The content is solely the responsibility of the authors and does not necessarily represent the official views of the granting agency. This research builds upon results first presented in the poster *Drums Alive Golden Beats Improves Brake Onset Time in Older Adults* (Poster Presentation) at the 19th Annual *CCTS Spring Conference in Lexington, KY, on Tuesday, April 9, 2024.* The poster was coauthored by Alaine E. Reschke‐Hernandez, Brittney Moshos, Peter Wright, Greg Walsh, Anne Graff, Carrie Ekins, Sarah Davey, Kiera Wilkinson, Nathan F. Johnson, and me.

## Conflicts of Interest

Carrie Ekins is the creator and owner of Drums Alive. She provided training for the Drums Alive protocol used in the study. She did not deliver the intervention nor participate in data collection, outcome assessment, or analysis. Austin S. Robinson and Anne Graff are certified Drums Alive instructors. They delivered the intervention but did not participate in data collection, outcome assessment, or analysis. The remaining authors declare no conflicts of interest.

## Data Availability

The data that support the findings of this study are available from the corresponding author upon reasonable request.
